# Speech Rhythms and Multiplexed Oscillatory Sensory Coding in the Human Brain

**DOI:** 10.1371/journal.pbio.1001752

**Published:** 2013-12-31

**Authors:** Joachim Gross, Nienke Hoogenboom, Gregor Thut, Philippe Schyns, Stefano Panzeri, Pascal Belin, Simon Garrod

**Affiliations:** 1 Institute for Neuroscience and Psychology, University of Glasgow, Glasgow, United Kingdom; 2 Institute for Clinical Neuroscience and Medical Psychology, University of Düsseldorf, Düsseldorf, Germany; 3 Center for Neuroscience and Cognitive Systems, Istituto Italiano di Tecnologia @UniTn, Rovereto, Italy; New York University, United States of America

## Abstract

Cortical oscillations are likely candidates for segmentation and coding of continuous speech. Here, we monitored continuous speech processing with magnetoencephalography (MEG) to unravel the principles of speech segmentation and coding. We demonstrate that speech entrains the phase of low-frequency (delta, theta) and the amplitude of high-frequency (gamma) oscillations in the auditory cortex. Phase entrainment is stronger in the right and amplitude entrainment is stronger in the left auditory cortex. Furthermore, edges in the speech envelope phase reset auditory cortex oscillations thereby enhancing their entrainment to speech. This mechanism adapts to the changing physical features of the speech envelope and enables efficient, stimulus-specific speech sampling. Finally, we show that within the auditory cortex, coupling between delta, theta, and gamma oscillations increases following speech edges. Importantly, all couplings (i.e., brain-speech and also within the cortex) attenuate for backward-presented speech, suggesting top-down control. We conclude that segmentation and coding of speech relies on a nested hierarchy of entrained cortical oscillations.

## Abstract 2

A neuroimaging study reveals how coupled brain oscillations at different frequencies align with quasi-rhythmic features of continuous speech such as prosody, syllables, and phonemes.

## Author Summary

Continuous speech is organized into a nested hierarchy of quasi-rhythmic components (prosody, syllables, phonemes) with different time scales. Interestingly, neural activity in the human auditory cortex shows rhythmic modulations with frequencies that match these speech rhythms. Here, we use magnetoencephalography and information theory to study brain oscillations in participants as they process continuous speech. We show that auditory brain oscillations at different frequencies align with the rhythmic structure of speech. This alignment is more precise when participants listen to intelligible rather than unintelligible speech. The onset of speech resets brain oscillations and improves their alignment to speech rhythms; it also improves the alignment between the different frequencies of nested brain oscillations in the auditory cortex. Since these brain oscillations reflect rhythmic changes in neural excitability, they are strong candidates for mediating the segmentation of continuous speech at different time scales corresponding to key speech components such as syllables and phonemes.

## Introduction

A large number of invasive and non-invasive neurophysiological studies provide converging evidence that cortical oscillations play an important role in gating information flow in the human brain, thereby supporting a variety of cognitive processes including attention, working memory, and decision-making [1]–[3]. These oscillations can be hierarchically organised. For example, the phase of (4–8) Hz theta oscillations can modulate the amplitude of (30–90 Hz) gamma oscillations; the phase of (1–2 Hz) delta oscillations can modulate the amplitude of theta oscillations [4]–[8].

Interestingly, speech comprises a remarkably similar hierarchy of rhythmic components representing prosody (delta band), syllables (theta band), and phonemes (gamma band) [9]–[12]. The similarity in the hierarchical organisation of cortical oscillations and the rhythmic components of speech suggests that cortical oscillations at different frequencies might sample auditory speech input at different rates. Cortical oscillations could therefore represent an ideal medium for multiplexed segmentation and coding of speech [9],[12]–[17]. The hierarchical coupling of oscillations (with fast oscillations nested in slow oscillations) could be used to multiplex complementary information over multiple time scales [18] (see also [19]) for example by separately encoding fast (e.g., phonemic) and slower (e.g., syllabic) information and their temporal relationships.

Previous studies have demonstrated amplitude and phase modulation in response to speech stimuli in the delta, theta, and gamma bands using electroencephalography (EEG)/magnetoencephalography (MEG) [13],[15],[20]–[25] and electrocorticography (ECOG) [26]–[29]. These findings support an emerging view that speech stimuli induce low-frequency phase patterns in auditory areas that code input information. Interestingly, these phase patterns seem to be under attentional control. For example, in the well known cocktail party situation, they code mainly for the attended stimulus [26],[30],[31]. Thus, brain oscillations have become obvious candidates for segmenting and parsing continuous speech because they reflect rhythmic changes in excitability [12].

This attractive model leaves three important points largely unresolved: First, a comprehensive account of how rhythmic components in speech interact with brain oscillations is still missing and it is uncertain if the previously reported hemispheric asymmetry during speech perception is also evident in a lateralized alignment of brain oscillations to continuous speech. Behavioural, electrophysiological, and neuroimaging studies [13],[15],[20],[23],[32] suggest that there is a relatively long integration window (100–300 ms, corresponding to the theta band) in the right auditory cortex and a relatively short integration window (20–40 ms, corresponding to the gamma band) in the left auditory cortex [14]. But it is unclear whether this differentiation is relevant for oscillatory tracking of speech. Second, it is unknown whether cortical brain oscillations are hierarchically coupled during perception of continuous speech. This is of particular interest because hierarchically coupled brain oscillations could represent hierarchically organised speech components (prosody, syllables, phonemes) at different temporal scales. Third, it is unclear how oscillatory speech tracking dynamically adapts to arrhythmic components in speech. If brain oscillations implement a universal mechanism for speech processing they should also account for variations or breaks in speech rhythmicity, so that the phase of low-frequency oscillations aligns to (quasi-periodic) salient speech events for optimal processing.

Here, we addressed these three points using continuous speech and analysis based on information theory. Importantly, all three points were investigated for intelligible and unintelligible (backward played) speech. We analysed the frequency-specific dependencies between the speech envelope and brain activity. We also analysed the dependencies between cortical oscillations across different frequencies. We first hypothesised that a multi-scale hierarchy of oscillations in the listener's brain tracks the dynamics of the speaker's speech envelope—specifically, preferential theta band tracking in the right auditory cortex and gamma band tracking in the left auditory cortex. Second, we asked whether speech-entrained brain oscillations are hierarchically coupled and if so how that coupling is modulated by the stimulus. Third, we asked whether phase of low-frequency brain oscillations (likely indicating rhythmic variations in neural excitability) in the auditory cortex coincide with and adapt to salient events in speech stimuli.

**Figure 1:**
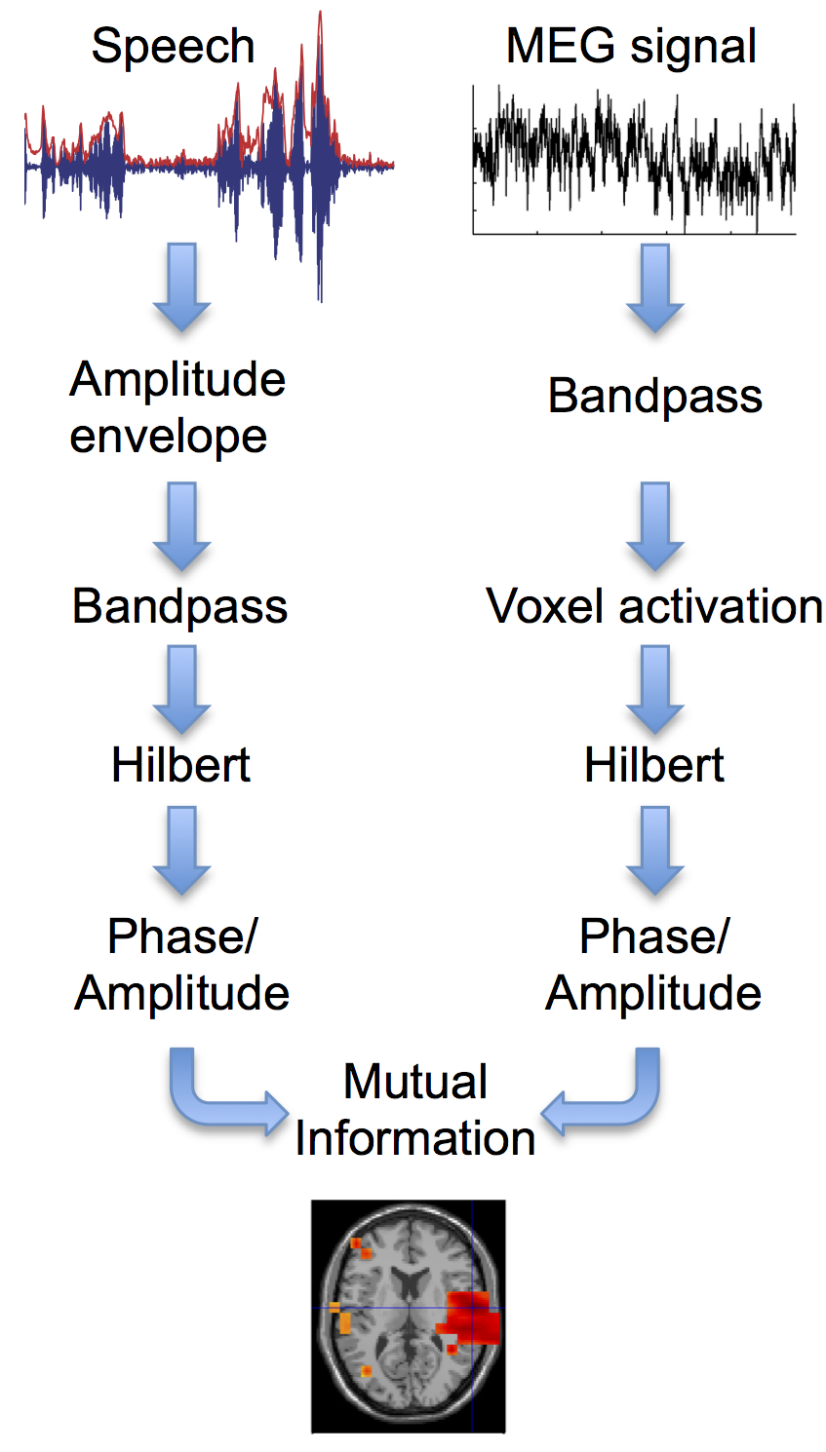
Mutual information analysis. The broadband amplitude envelope is computed for the speech signal. For each frequency band speech envelope and MEG signals are bandpass filtered and activation time series are computed for each voxel in the brain. Phase and amplitude time series are computed from the Hilbert transform for speech and voxel time series and subjected to MI analysis. MI is computed between speech signal and time series for each voxel leading to a tomographic map of MI. Group statistical analysis is performed on these maps across all 22 participants.

We presented a 7-min long continuous story binaurally to 22 participants while recording neural activity with MEG (“story” condition). As a control condition the same story was played backwards (“back” condition). We used mutual information (MI) to measure all dependencies (linear and nonlinear) between the speech signal and its encoding in brain oscillations [33],[34]. We did so in all brain voxels for frequencies from 1 to 60 Hz and for important interactions (phase-phase, amplitude-amplitude, cross-frequency phase-amplitude, and cross-frequency amplitude-phase, see Figure 1 and Materials and Methods). This resulted in frequency specific functional brain maps of dependencies between the speech envelope and brain activity. Similar analysis was performed to study dependencies between brain oscillations within cortical areas but across different frequency bands.

Our results reveal hierarchically coupled oscillations in speech-related brain areas and their alignment to quasi-rhythmic components in continuous speech (prosody, syllables, phonemes), with pronounced asymmetries between left and right hemispheres. Edges in the speech envelope reset oscillatory low-frequency phase in left and right auditory cortices. Phase resets in cortical oscillations code features of the speech edges and help to align temporal windows of high neural excitability to optimise processing of important speech events. Importantly, we demonstrate that oscillatory speech tracking and hierarchical couplings significantly reduce for backward-presented speech and so are not only stimulus driven.

## Results

### Oscillatory Speech Tracking Relies on Two Mechanisms

**Figure 2:**
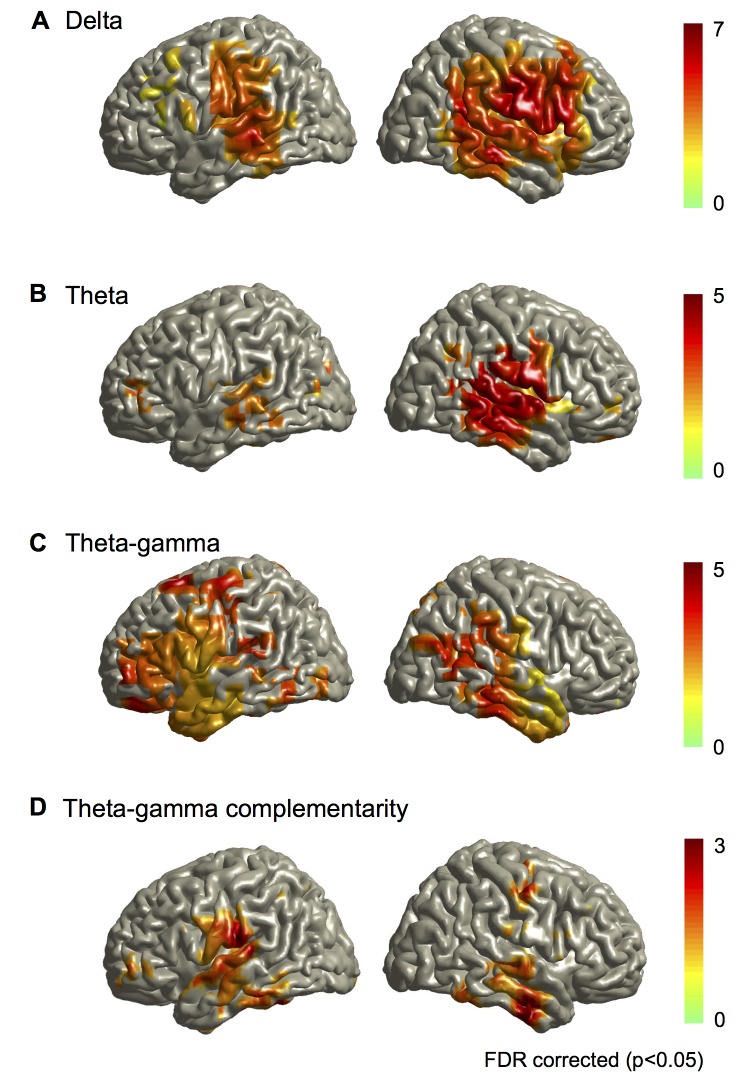
Mutual information group statistics. All statistical maps are thresholded at *p* = 0.05 (FDR corrected) and colourbars show *t*-values. (A) Group statistical map of MI between speech phase and phase of brain activity in the delta frequency band (1–3 Hz) for the statistical contrast story versus back (see Figure S1 for corresponding map using PLV). (B) Group statistical map of MI between speech phase and phase of brain activity in the theta frequency band (3–7 Hz) for the statistical contrast story versus back (see Figure S1 for corresponding map using surrogate data). (C) Group statistical map of MI between 3–7 Hz theta phase in speech signal and 35–45 Hz gamma amplitude in brain activity for the contrast story versus back. (D) Complementarity between theta phase and gamma amplitude. Mutual information between theta phase in speech and theta phase in brain activity was computed with and without corresponding gamma amplitude signal. The statistical map shows significantly increased MI when gamma amplitude is used in addition to theta phase.

We first asked whether there is phase-locking between rhythmic changes in the speech envelope and corresponding oscillatory brain activity. Whereas most previous studies quantify phase-locking to stimulus onset across repeated presentations of the same stimulus, here we studied phase-locking over time directly between speech envelope and brain oscillations. To do this, we compared the phase coupling between the speech and oscillatory brain activity (in 1 Hz steps between 1 and 60 Hz) in two conditions: story and back. Figure 2 summarizes the results. First, MI revealed a significantly stronger phase coupling between the speech envelope and brain oscillations in the story compared to back conditions in the left and right auditory cortex in delta (1–3 Hz) and theta (3–7 Hz) frequency bands (group statistics, *p*<0.05, false discovery rate [FDR] corrected, see Figure 2A and 2B). These results confirm that low-frequency rhythmic modulations in the speech envelope align with low-frequency cortical oscillations in auditory areas (using phase-locking value (PLV) instead of MI and contrasting story with surrogate data lead to virtually identical results, see Figure S1).

To test for other couplings between the speech and cortical oscillations, we also computed MI between the amplitude of the speech and the amplitude of cortical oscillations and between the amplitude of the speech and the phase of cortical oscillations for each frequency between 1 and 60 Hz. These computations revealed no significant dependencies. Finally, we flipped the computations around, to test whether the phase of the speech envelope modulated the amplitude of cortical oscillations. Again, we carried out this computation across frequencies, for all combinations between 1 and 60 Hz and found one significant phase-amplitude coupling. Figure 2C illustrates that low-frequency changes in the speech envelope (at 3–7 Hz) modulate the amplitude of 35–45 Hz gamma activity in both auditory cortices significantly more strongly in the story compared to the back condition.

In sum, this comprehensive analysis revealed two distinct speech tracking mechanisms in the brain. First, low-frequency speech modulations entrain (that is, align the phase of) delta and theta oscillations in the auditory cortex. Second, low-frequency speech modulations also entrain the amplitude dynamics of gamma oscillations. Both tracking mechanisms are especially sensitive to intelligible speech because the effects are stronger for the story than the back condition. Since the theta phase of the speech envelope is coupled to both, the theta phase (Figure 2B) and gamma amplitude (Figure 2C) of auditory brain oscillations, we investigated if both these signals represent the same or different information about the speech stimulus. Again, we performed the analysis within an information-theoretic framework based on that of Ince et al. [35]. Specifically, we investigated whether the information about speech in the theta phase of auditory oscillations is similar or complementary to that carried by gamma power. We computed whether gamma amplitude adds significant mutual information about the speech envelope over and above the information carried by the theta phase of brain activity (see Materials and Methods section for details). The analysis revealed that gamma amplitude does add significant complementary information to theta phase. Gamma amplitude adds on average 23% (±7 standard error of the mean [SEM]) to theta phase information. Figure 2D illustrates this complementarity and shows how it is particularly pronounced for the left auditory cortex. This suggests that each mechanism is partly independent of the other and thus can capture complementary information about the stimulus.

### Oscillatory Speech Tracking Is Lateralised

**Figure 3:**
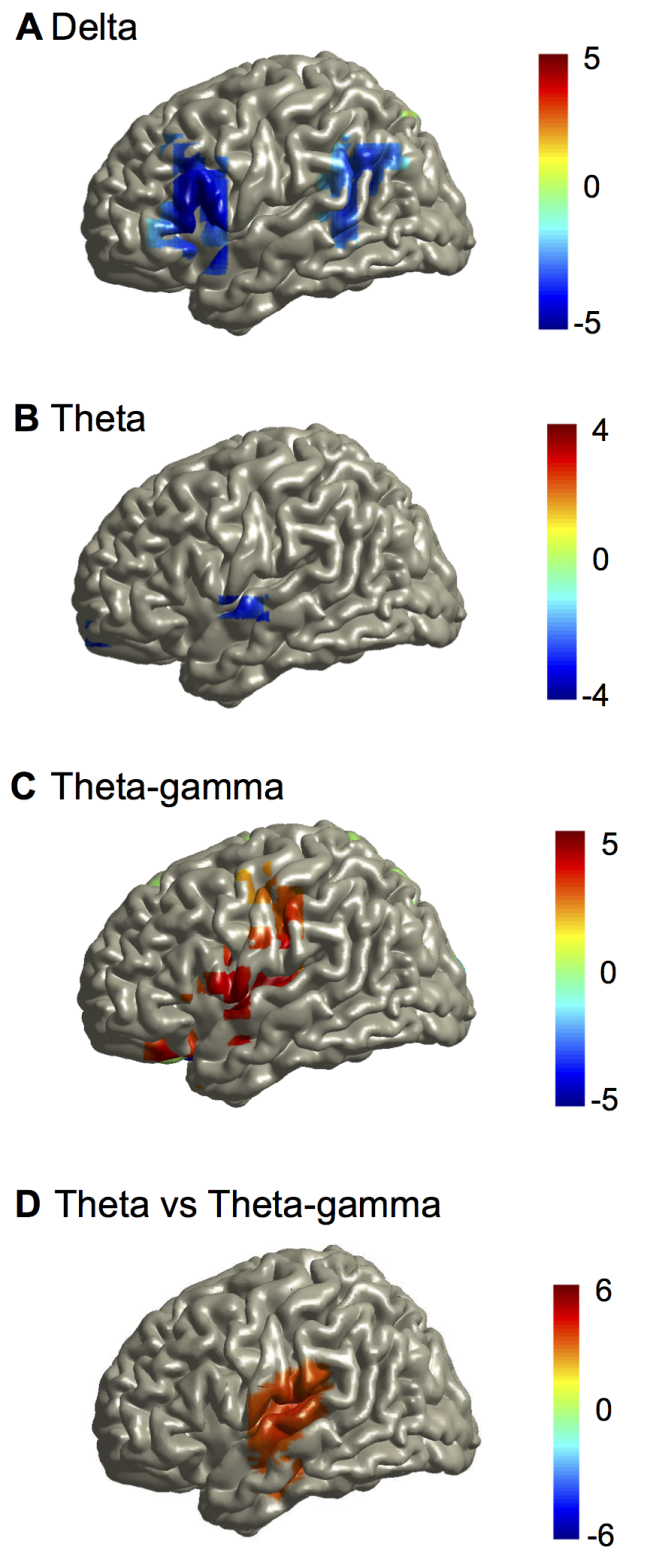
Mutual information group statistics of lateralisation in the story condition. All maps show *t*-statistics of lateralisation index (left−right)/(left+right) of mutual information. Red colours indicate lateralisation to the left cortical areas. Only the left hemisphere is shown because results are redundant in the right hemisphere. (A) Group statistical map of lateralisation of delta band MI (corresponding to Figure 2A). (B) Group statistical map of lateralisation of theta band MI (corresponding to Figure 2B). (C) Group statistical map of lateralisation of theta phase to gamma amplitude coupling (corresponding to Figure 2C). (D) Group statistical map comparing theta phase to gamma-amplitude lateralisation versus theta phase lateralisation. Maps are thresholded at *p* = 0.05 (FDR corrected).

Next we statistically tested for possible lateralisation of these different tracking mechanisms. The analysis was based on FDR-corrected dependent samples' *t*-tests of MI values for corresponding voxels in the left and the right hemisphere for the story condition. Interestingly, although present in both left and right hemisphere (Figure 2A and 2B), delta and theta phase-locking to speech was significantly stronger in the right (Figure 3A and 3B). Lateralisation maps also revealed a spatial dissociation whereby delta MI was right-lateralised in frontal and parietal areas whereas theta MI was only right-lateralised in superior temporal areas. In contrast, gamma amplitude tracking showed the opposite lateralisation with stronger coupling to speech in the left as compared to the right auditory cortex (Figure 3C). Finally, we compared lateralisation of theta phase tracking to lateralisation of gamma-amplitude tracking for the story condition. The statistical map shows significantly higher lateralisation for theta phase tracking in the right auditory cortex but significantly higher lateralisation for gamma amplitude tracking in the left auditory cortex (Figure 3D). We further confirmed these group results for single participants. A similar lateralisation pattern was seen in 17 out of 22 participants corroborating the group statistics (Figure S2). Mutual information values (mean and SEM) for the left and right auditory cortex are displayed as bar plots in Figure S3 for all conditions illustrating the lateralisation patterns.

This analysis revealed differential hemispheric preference for the two coupling mechanisms. Whereas right hemisphere areas showed stronger low-frequency phase coupling to the speech envelope, left hemisphere areas showed stronger high-frequency amplitude coupling to the speech envelope.

### Oscillatory Speech Tracking Mechanisms Depend on a Nested Hierarchy of Brain Oscillations

**Figure 4:**
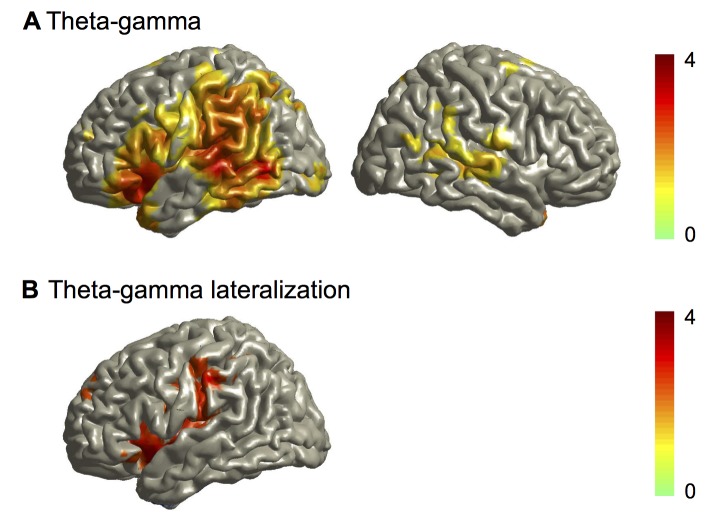
Group statistics of cross-frequency coupling. (A) Statistical map of difference between story and back condition for mutual information between theta phase and gamma amplitude. (B) Statistical map of lateralisation of mutual information between theta phase and gamma amplitude for the story condition.

This delta and theta phase coupling together with gamma amplitude coupling suggests that the brain oscillations might be nested [4]. To test for this cross-frequency coupling we computed the mutual information between the theta phase and gamma amplitude of each voxel across the 7-min dataset. By contrast to the analysis shown in Figure 2C, both the theta phase and the gamma amplitude were derived from the same voxel. The resulting mutual information map for each participant quantifies cross-frequency coupling of theta phase and gamma amplitude in each voxel. As before, we performed group statistics on the individual mutual information maps to identify significant differences between the story and back condition. Figure 4A shows significantly increased cross-frequency coupling (theta phase and gamma amplitude) for the story condition compared to the back condition both in bilateral auditory areas and in language areas of the left hemisphere.

Lateralisation analysis revealed that the modulation of gamma amplitude by theta phase is stronger in the left compared to right hemisphere (Figure 4B).

We performed the same analysis for cross-frequency coupling between delta phase and theta amplitude. The statistical difference map between the story and the back condition showed significant effects in bilateral temporal areas (Figure S4A) with lateralisation to left hemisphere (Figure S4B) but these effects were not as strong as those for the theta-gamma coupling.

In summary, these results indicate that oscillatory speech tracking is supported by a nested hierarchy of oscillations at delta, theta, and gamma frequencies and that these cross-frequency interactions are stronger for intelligible than for unintelligible speech.

### Phase Resets of Auditory Brain Oscillations by Speech Edges Improve Speech Tracking

At this juncture, it is important to note that speech, though rhythmic, is not strictly periodic: it comprises discontinuities and changes in syllable rate and duration. Any cortical speech tracking mechanism must be able to track these irregularities. We predicted that temporal edges in the speech envelope [36] should induce phase resets in the cortical oscillations tracking the speech thereby enhancing tracking. Here, we focussed on the theta band phase-locking because of its relation to the syllable rate.

**Figure 5:**
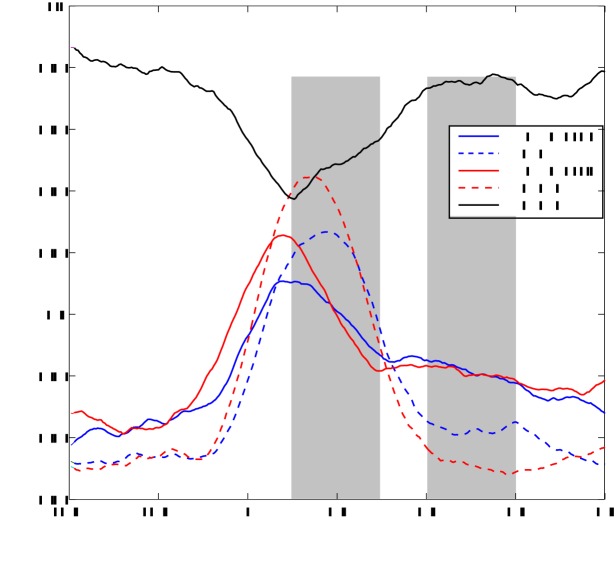
Phase-locking value in the auditory cortex time-locked to temporal speech edges. Phase-locking in theta frequency band between low-frequency speech envelope and the left (PLV speech L, blue solid line) and right (PLV speech R, red solid line) auditory cortex is shown following edge onset at 0 ms. Dashed lines show phase-locking across trials (regardless of speech signal) timelocked to edge onset for left (PLV L, blue dashed line) and right (PLV R, red dashed line). The black line represents phase-locking between the left and right auditory cortex.

We used a thresholding algorithm to identify 254 separate temporal edges in the continuous stimulus (see Materials and Methods for details). We then computed theta-band phase-locking between auditory theta activity and the theta phase of speech envelope time-locked to these edges. This quantifies the alignment between both signals as in Figure 2B but now time-locked to temporal edges. Figure 5 shows increased alignment between brain oscillations and speech envelope in the left (blue solid line) and the right (red solid line) auditory cortex following edges. *t*-Tests revealed significant (*p*<0.05) increase of phase-locking in an early (100–300 ms) and late (400–600 ms) time window compared to baseline (−200 to 0 ms).

To measure the extent to which this increase can be explained by a stereotypical edge-evoked response we computed phase-locking of auditory theta activity across trials time-locked to edge onset (dashed lines). This measure captures the evoked response to edge onset. As expected, this evoked response (dashed lines) increased following edge onset with a similar dynamics as the phase-locking to speech (solid lines). But importantly, phase-locking to speech (solid lines) is significantly stronger in the late time window than phase-locking to edge onset (dashed lines) (*t*-test, *p*<0.05). This demonstrates that speech continuously entrains brain rhythms beyond a stereotypical short-lived phase reset evoked by edges.

Finally, we computed the phase-locking between left and right auditory theta activity (Figure 5, black line). This measure quantifies the temporal coordination between both auditory cortices in the theta band. Interestingly, the increased phase alignment to speech coincided with a significant reduction of phase-locking between both auditory cortices in the early window. One interesting possibility is that this reduction in phase-locking reflects the more sensitive tracking of speech theta rhythms in the right auditory cortex compared to the left. Indeed, phase-locking to speech is significantly stronger in right than in the left auditory cortex from 50–100 ms (*t*-test, *p*<0.05). This could indicate that phase resetting in the left hemisphere is partly driven by the right auditory cortex.

Overall, the results confirmed our prediction. Edges in speech increased the alignment of auditory theta oscillations to the speech envelope and this increase outlasted the standard evoked response to edge onset. In addition, speech edges caused a significant transient decoupling of both auditory cortices.

### Oscillatory Speech Tracking Optimises Sampling of The Speech Signal

**Figure 6:**
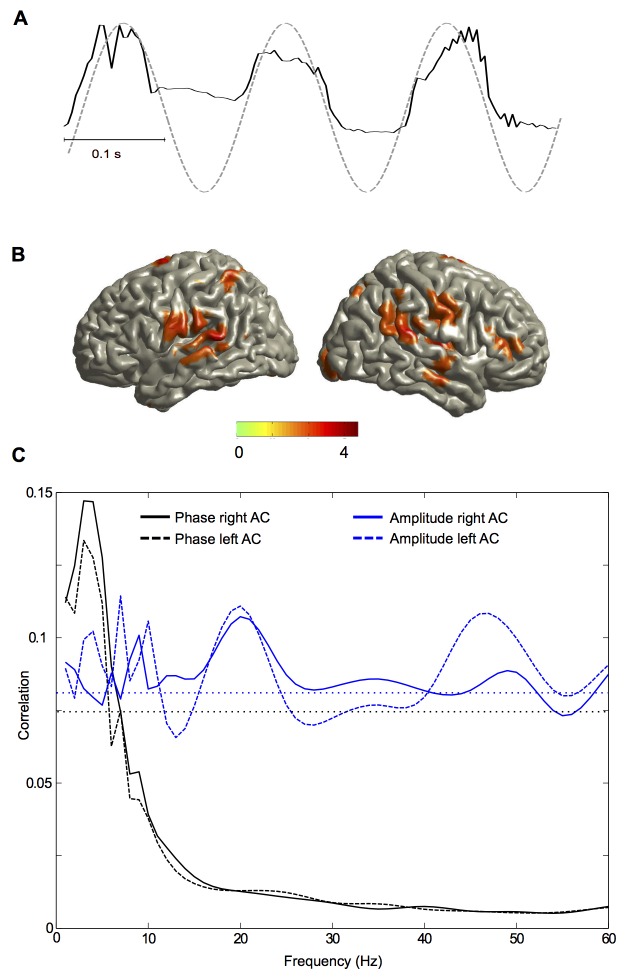
Oscillatory speech sampling. (A) Speech envelope (black line) and cosine of theta phase of the right auditory cortex of one participant for one trial. (B) The spatial distribution of significant correlation between low-frequency (3–7 Hz) phase and speech envelope (*p*<0.05, FDR corrected). The statistical map shows *t*-values of the statistical contrast between correlations for the story condition and trial-shuffled surrogate data. (C) Spectrum of cross-correlation between oscillations in the left and right auditory cortex and speech envelope. Black lines correspond to correlations based on the cosine of phase and blue lines to correlations based on amplitude. Solid lines represent the right auditory cortex and dashed lines represent the left auditory cortex. Horizontal dotted lines show 95th percentile of chance distribution of the maximum across frequencies obtained from shuffled data for phase (black) and amplitude (blue).

Since oscillations represent rhythmic fluctuations in the excitability of neural populations we hypothesised that phase-locking (assisted by phase resetting) between the speech envelope and low-frequency oscillations in the auditory cortex implements a mechanism for efficient sampling and segmentation of speech [12],[31]. To directly test this sampling hypothesis, we measured the correlation between each cortical oscillatory band between 1 and 60 Hz and the speech envelope for the 254 trials identified in the previous analysis. Figure 6A illustrates this analysis for a sample taken from one individual. The black line shows the speech envelope for a given trial and the dashed line shows the cosine of theta phase in the right auditory cortex for this participant. In the full analysis we computed the cross-correlation for each brain voxel and for each of the 254 trials (defined as the 500 ms following an onset) and then averaged the absolute correlation across trials, for each oscillatory band independently. To account for the different tracking mechanisms identified above (phase tracking and amplitude tracking), we computed two correlations. First, we correlated the cosine of the phase of cortical oscillations with the speech envelope. Second, we correlated the amplitude of cortical oscillations with the speech envelope. For comparison, we also computed these correlations after randomly shuffling the trial order of the speech envelope.

Figure 6B shows significantly higher correlations in left and right auditory areas for low-frequency phase oscillations compared with the shuffled condition. Figure 6C presents the spectral profile of correlation for the left and right auditory cortex. At frequencies below 10 Hz the phase of auditory oscillations shows higher correlations with the speech envelope than does amplitude. Above 10 Hz this pattern is reversed. Interestingly the correlation based on amplitude (blue lines) shows a peak at 40–50 Hz in agreement with Figure 2C. An additional peak is evident at about 20 Hz. Speech sampling by phase in the delta and theta band in the left and right auditory cortex is significantly higher for the story compared to the back condition (and also compared to trial-shuffled data, paired *t*-tests, all *p*<0.05). Speech sampling by amplitude in the gamma band is significantly higher for the story compared to the back condition in the left auditory cortex (and compared to trial-shuffled data in both auditory cortices). Although the pattern of lateralisation was overall consistent with Figure 3, the difference in lateralisation did not reach significance. This is probably because this correlation measure is less sensitive than the mutual information analysis on the band-pass filtered speech envelope reported in Figure 3.

These results indicate that temporal edges in speech amplitude induce modulations in low-frequency phase and high-frequency amplitude dynamics of brain oscillations that align windows of high neural excitability to salient speech events. Importantly, this alignment is not caused by an identical phase resetting for all edges because shuffling the speech trials reduces the correlation. We predicted that edge-specific phase resets coding stimulus features (e.g., edge amplitude) cause this trial-specific alignment. We tested this hypothesis by sorting our previously identified 254 trials by maximum amplitude of speech envelope in the 200 ms window after onset. For each participant we computed in the left and right auditory cortex the theta phase at 100 ms after onset and correlated both quantities using circular correlation [37]. Significant correlation was observed in the left and right auditory cortex (Figure S5).

Together, these results demonstrate that the phase of low-frequency cortical oscillations and the amplitude of high-frequency oscillations align to trial-specific speech dynamics, adapting to variations of speech over time. This trial-specific alignment suggests that oscillatory windows of high excitability sample salient speech components. Our analysis on the continuous data (Figures 4 and S4) has demonstrated a nested hierarchy of oscillations in the auditory cortex with stronger cross-frequency coupling for intelligible speech compared to unintelligible speech. Since edges enhance oscillatory speech tracking we hypothesised that edges also increase this cross-frequency coupling. We tested this hypothesis in our final analysis.

### Speech Edges Increase Cross-Frequency Coupling

We first characterised the spatial distribution of edge-induced changes in cross-frequency coupling by computing coupling of gamma amplitude to theta phase in all brain voxels. We then computed the full cross-frequency coupling matrix separately for the left and the right auditory cortex.

**Figure 7:**
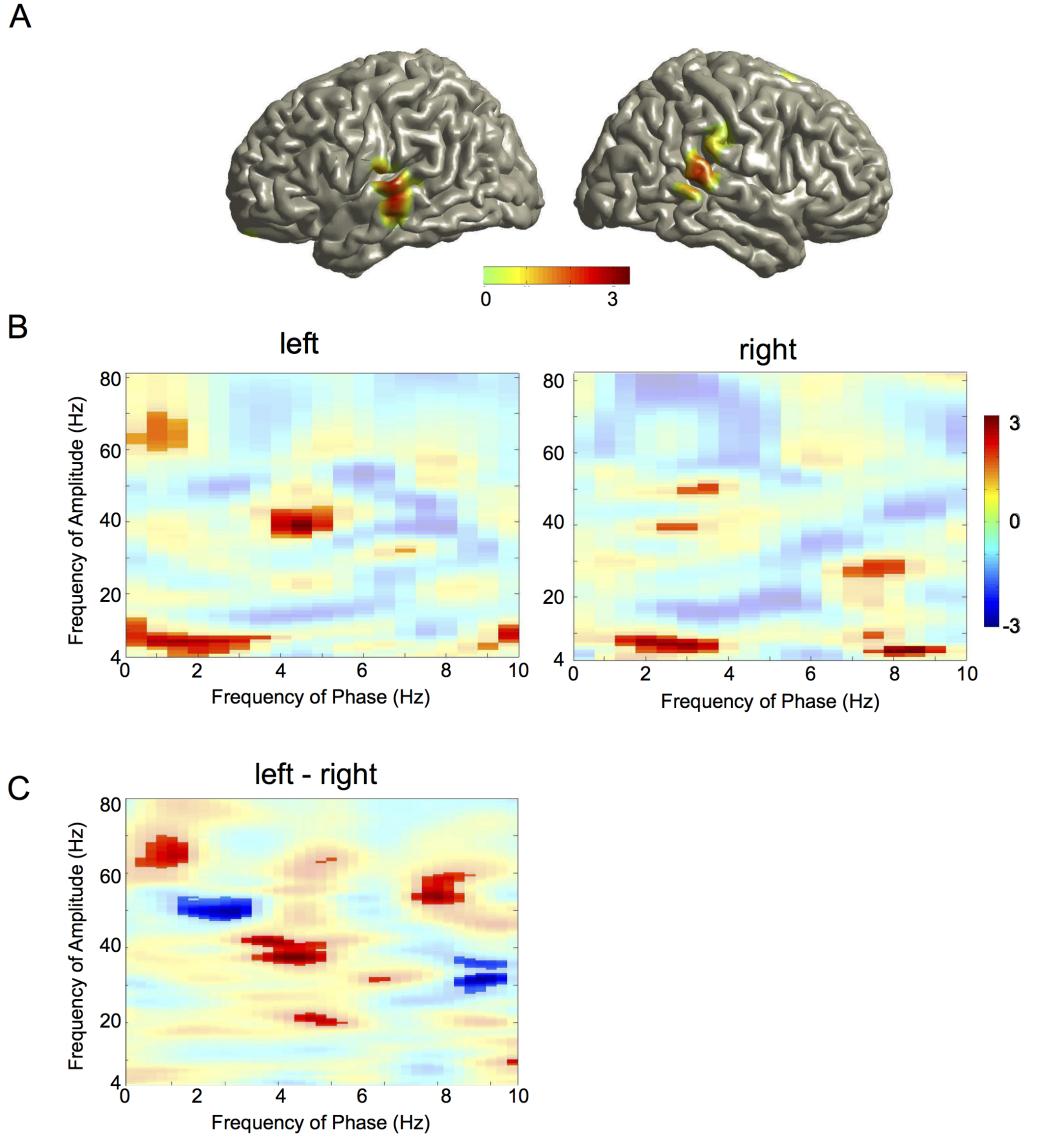
Cross-frequency phase-amplitude coupling. (A) Spatial distribution of theta phase to gamma amplitude coupling. Group statistical map of difference between story and back condition thresholded at *p* = 0.05 (FDR corrected). Colour code represents *t*-values. (B) Spectral distribution of phase-amplitude coupling in the auditory cortex. Cross-frequency phase-amplitude coupling quantified with MI is shown for the left and right auditory cortex. Pixels with significant difference between story and surrogate condition are displayed as opaque. (C) Lateralisation of cross-frequency phase-amplitude coupling. Pixels with significant lateralisation are displayed as opaque. Positive *t*-values indicate left-lateralized effects.

As before, we used MI to analyze cross-frequency oscillatory coupling (as in Figure 4A) but now time-locked to edges. For each brain voxel, across all 254 trials we computed a *t*-statistic of MI between theta phase and gamma amplitude for the two 500 ms windows preceding and following speech onset. Since this computation is based on the difference between post-stimulus and pre-stimulus data it captures the edge-induced changes of cross-frequency coupling. We performed the computation for both the story and back condition. As in Figure 2 we submitted individual maps to dependent samples *t*-test (story versus back condition) with randomisation-based FDR correction. Group *t*-maps are displayed with thresholds corresponding to *p*<0.05 (FDR-corrected). Figure 7A shows the spatial distribution of theta phase to gamma-amplitude coupling. Left and right auditory areas show a significant difference of edge-induced changes in cross-frequency coupling between the story and back condition.

The second analysis used the left and right auditory cortex as regions of interest to compute the full cross-frequency coupling matrix. Here, we computed MI as before but now for all combinations of phase (1–10 Hz) and amplitude (4–80 Hz). We computed group *t*-statistics for the difference between the story condition and surrogate data (significant pixels are opaque, see Materials and Methods). Both left and right auditory cortices show a frequency-specific coupling of theta phase to gamma amplitude and in addition a frequency-specific coupling of delta phase and theta amplitude (Figure 7B). Both effects are significantly stronger (*t*-test, *p*<0.05) in the story condition compared to the back condition, demonstrating a more precise hierarchical nesting of cortical oscillations for intelligible than unintelligible speech.

Finally, we studied lateralisation of the cross-frequency coupling shown in Figure 7B. The results in Figure 7C demonstrate a significant lateralisation of theta-gamma coupling to the left auditory cortex.

## Discussion

Our results provide direct evidence for the hypothesis that a listener's brain oscillations segment and encode continuous speech in a frequency-specific manner. This suggests that these oscillations play a functional role in efficient sensory sampling. MI analysis reveals alignment of low-frequency phase and high-frequency amplitude to the speech envelope that is frequency specific, shows hemispheric asymmetry, and is modulated by intelligibility (i.e., enhanced for story compared to back condition). The low-frequency phase alignment is preserved over time by transient events in the stimulus (edges) that lead to phase adjustments. These phase adjustments are stimulus specific and depend on the amplitude of transient events (and likely other features of the stimulus). Interestingly, brain activity in the three observed frequency bands is hierarchically coupled. This cross-frequency coupling is increased following edge onset and the increase is stronger for speech than for reverse speech.

### Spatio-Spectral Characteristics of Speech Entrainment

We observed phase alignment between low-frequency components of the speech envelope and brain activity in the delta and theta band. No consistent phase-phase coupling was observed for frequencies higher than 10 Hz. Previous studies have shown that speech envelope frequencies below 10 Hz are important for intelligibility [38]. Indeed, delta and theta frequencies match the rhythmicity of important temporal structures in continuous speech. Slow speech envelope variations (0.3–1 s, delta band) represent prosody whereas syllables tend to occur at a rate of about 3–7 Hz in normal speech [9],[10]. These components are known to modulate oscillatory phase and amplitude dynamics in the auditory cortex [12]. Our study investigated the underlying mechanisms by using information theory to comprehensively quantify how the phase and amplitude of different frequency components of the speech envelope affect the phase and amplitude of different cortical brain oscillations.

We reported two different mechanisms. First, the low-frequency phase in the speech envelope entrains the low-frequency phase of brain oscillations in delta and theta frequency bands. The specific entrainment patterns support the idea that delta and theta bands are qualitatively different [25]. Phase coupling in the delta band extends more towards right frontal areas compared to theta phase coupling and both frequencies show different spatial lateralisation patterns (Figure 3). This indicates selective engagement of different areas for processing the different quasi-rhythmic components of the stimulus. Interestingly, significant right-lateralisation was evident in the delta band in frontal, posterior temporal, and parietal areas but not in primary auditory areas (in contrast to the theta band). These results are consistent with previous findings that right temporal and frontal brain areas are involved in prosodic processing [24],[39]. Bilateral auditory areas show significant theta phase entrainment to the speech envelope. This effect is significantly lateralised to the right hemisphere and confirms previous findings [20],[23].

The second mechanism revealed in our analysis is the alignment of gamma-amplitude modulations to the theta phase of the speech envelope in bilateral temporal, frontal, and parietal areas with lateralisation to the left hemisphere. Taken together, the auditory cortex showed right-lateralisation for theta phase entrainment and left-lateralisation for gamma amplitude entrainment. These results support the asymmetric sampling in time (AST) model [12],[14],[40] (but see [41]) that suggests a right-hemispheric preference for long temporal integration windows of 100–300 ms (corresponding to theta band) and a left-hemispheric preference for short temporal integration windows of about 20–40 ms (corresponding to gamma frequencies). Indeed, this view is supported by studies of phase consistency in the theta band [20],[23] and of oscillatory power in the gamma band [13],[42],[43]. Our results demonstrate a direct effect of specific speech components (low-frequency phase of speech envelope) on oscillatory brain activity and show significant lateralisation consistent with the AST-model. Interestingly, this coupling of brain oscillations to speech rhythms is supported by a hierarchical coupling of brain oscillations across frequencies. Delta phase modulates theta amplitude and theta phase modulates gamma amplitude and this modulation is stronger for intelligible compared to unintelligible speech. The hierarchically coupled oscillations could represent speech components (prosody, syllables, phonemes) in parallel at different timescales while preserving their mutual relationships.

All entrainment effects were identified in a statistical contrast between the story and the back condition. This is important because it demonstrates that these entrainments are not just unspecific stimulus-driven effects but that they are modulated by intelligibility of the stimulus. A previous study [44] did not find entrainment differences between the two conditions. This might be explained by the fact that their stimulus material consisted only of three sentences across the whole study leading to learning effects even for the reversed speech. Also, the specific task used in that paper did not require comprehension and therefore might have masked differences between the speech and reversed speech condition. Reverse speech is often used as a control condition in speech experiments [44]–[46] since the physical properties of the stimulus are preserved. Especially, rhythmic components in the speech stimuli are still present in reversed speech (although the quasi-periodicity of rhythmic components in speech will lead to some changes in the oscillatory dynamics of reversed speech). The enhanced entrainment observed in the story condition is therefore likely due to top-down mechanisms that have been previously shown to modulate activity in the auditory cortex during processing of degraded speech [47],[48] or speech in noise [49]. These mechanisms could lead to changes in oscillatory phase dynamics [26],[50],[51]. We expect that within sentences, paragraphs, and over the entire course of the story participants will predict upcoming words and salient auditory events. This content-based prediction in the story condition seems to affect phase entrainment in early sensory areas [22],[52]–[54].

### Phase Resetting and Oscillatory Speech Sampling

Our study supports emerging models of speech perception that emphasise the role of brain oscillations [9],[12]. Hierarchically organised brain oscillations may sample continuous speech input at rates of prominent speech rhythms (prosody, syllables, phonemes) and represent a first step in converting a continuous auditory stream to meaningful internal representations. Our data suggest that this step of sparsening the sensory representation occurs in parallel computations both in frequency (as multiplexed oscillations) and in the left and right hemisphere [40] albeit with lateralised preference for different time scales.

Our results indicate that sharp large-amplitude transients (edges) in speech reset oscillations in the auditory cortex with important consequences. First, these resets increase the alignment between auditory oscillations and the speech envelope (Figure 6). This is important to re-align brain oscillations to speech after breaks. Second, this increase in alignment accounts for variations in continuous speech because randomly shuffling the speech signal across trials reduces the alignment. Since each trial represented a different segment of the continuous story this finding shows that brain oscillations are dynamically aligned to the time-varying dynamics of speech. Third, cross-frequency coupling between auditory oscillations increases following edges thereby enhancing precision of multi-scale nested dependencies. Fourth, temporal edges lead to a transient decoupling of the left and right auditory cortex that could be caused by a differential phase reset in both cortices and could indicate sensitivity to different acoustic properties of the stimulus.

In the rat auditory cortex, increases in sound power in the frequency band matching the tonotopy of the considered location lead to large depolarizing currents in the input layers that reset intrinsic oscillations to an “excitable” phase [55] (see also [56],[57]). It is therefore conceivable that our observed phase resets to edges realigns the internal temporal reference frame to the sensory input to optimally sample relevant information at oscillatory phases of high excitability. This phase reset is stimulus dependent because correlation with speech is reduced for trial-shuffled data (Figure 6) and because phase after edge-onset codes the amplitude of this edge (Figure S5). This coding of peak stimulus amplitude (and possible other features) in low-frequency phase could explain the previously reported classification of stimulus identity from low-frequency phase dynamics [58],[59]. The stimulus-specific phase resetting could be an important mechanism for aligning time windows of high neural excitability to salient stimulus events because of similar time constants in speech and brain dynamics. The importance of edges for speech entrainment was very recently shown by Doelling et al. [60]. By manipulating the speech envelope they demonstrated that edges enhance speech entrainment and intelligibility.

In summary, we report a nested hierarchy of auditory oscillations at multiple frequencies that match the frequency of relevant linguistic components in continuous speech. These oscillations entrain to speech with differential hemispheric preference for high (left) and low (right) frequencies. Our results indicate that temporal edges in speech increase first the coupling between auditory oscillations across frequency bands and, second, their coupling to the speech envelope.

We can only speculate about the nature of the observed phase/amplitude alignments. Most likely the alignments are caused by a combination of modulatory and evoked effects [55],[56] where stimulus-driven activity is top-down modulated via ongoing oscillatory activity [30],[61]. In this framework oscillatory activity is a mechanism for attentional selection and flexible gating of information from primary sensory areas.

Finally, going beyond speech perception, the entrainment of hierarchically organized oscillations between speaker and listener may well have a more general role in interpersonal communication [62],[63].

## Materials and Methods

### Participants and Recording

22 healthy, right-handed volunteers participated in the study (11 males; age range 19–44 years, mean 27 years). All participants provided informed written consent and received monetary compensation for their participation. The study was approved by the local ethics committee (University of Glasgow Faculty of Information and Mathematical Sciences) and conducted in conformity with the Declaration of Helsinki.

MEG recordings were obtained with a 248-magnetometers whole-head MEG system (MAGNES 3600 WH, 4-D Neuroimaging) at 1,017 Hz sampling rate.

The analysis of the MEG signal was performed using the FieldTrip toolbox [64], the Information-Theory Toolbox [33], and in-house MATLAB code according to recently published guidelines [65].

Stimuli have been previously used in an fMRI study [66]. The main stimulus consisted of a recording of a 7-min real-life story (“Pie-man,” told by Jim O'Grady at “The Moth” storytelling event, New York). The story was presented binaurally via a sound pressure transducer through two 5 m long plastic tubes terminating in plastic insert earpieces. Presentation was controlled with Psychtoolbox [67] under MATLAB. In addition to one standard presentation of the story (story), individuals also listened to the backward played story (back). Eye fixation was maintained throughout the experiment. Experimental conditions were recorded in randomised order.

### Analysis

#### Speech preprocessing

We computed the amplitude envelope of auditory signals following Chandrasekaran et al. [11]. Using the Chimera toolbox we constructed nine frequency bands in the range 100–10,000 Hz to be equidistant on the cochlear map [68]. Auditory stimuli were band-pass filtered in these bands using a fourth-order Butterworth filter (forward and reverse). Amplitude envelopes for each band were computed as absolute values of the Hilbert transform and averaged across bands to obtain a wide-band amplitude envelope that was used for all further analysis.

MEG signals were denoised with information from the reference sensors using the denoise_pca function in FieldTrip. Bad channels were excluded by visual inspection.

#### MEG-MRI co-registration

T1-weighted structural magnetic resonance images (MRIs) of each participant were co-registered to the MEG coordinate system using a semi-automatic procedure. Anatomical landmarks (nasion, left and right pre-auricular points) were manually identified in the individual's MRI. Initial alignment of both coordinate systems was based on these three points. Subsequently, numerical optimisation was achieved by using the ICP algorithm [69]. All region-of-interest analysis for the auditory cortex is based on the mean effect of all voxels in BA 41.

#### Source localisation

Individual head models were created from anatomical MRIs using segmentation routines in FieldTrip/SPM5. Leadfield computation was based on a single shell volume conductor model [70] using a 10 mm grid defined on the template (MNI) brain. The template grid was transformed into individual head space by linear spatial transformation.

Cross-spectral density was computed using Fast Fourier Transform on 1-s segments of data after applying Hanning window. For frequencies above 40 Hz spectral analysis was performed using multitaper (±5 Hz frequency smoothing [71]). Source localisation was performed using DICS [72]. Beamformer coefficients were computed sequentially for all frequencies from 1 to 60 Hz for the dominant source direction in all voxels with a regularisation of 7% of the mean across eigenvalues of the cross-spectral density matrix.

#### Mutual information

Dependencies between phase and amplitude of speech and MEG signal were all analysed in the common framework of information theory [73]. Specifically, MI between two signals was computed using the Information-Theory Toolbox [33]. MI measures how much knowing one signal reduces the uncertainty about the other signal. MI analysis was used because it captures both linear and non-linear dependencies (in contrast to coherence or correlation) and it affords the quantification of encoding by a range of sound and brain activity features (e.g., phase-phase, amplitude-amplitude, phase-amplitude, or cross-frequency encoding) within the same theoretic framework and on a common principled scale in units of bits.

First, frequency-specific brain activation time series were computed by applying the (frequency-specific) beamformer coefficients to the MEG data filtered in the same frequency band (fourth order Butterworth filter, forward and reverse, centre frequency ±1 Hz (or ±5 Hz for frequencies above 40 Hz). The broadband speech envelope was processed identically. Second, Hilbert transform was applied to the bandpass filtered data to compute phase or amplitude dynamics. Finally, MI was computed between the speech signal and brain signal for each voxel, frequency band, and for all combinations of signals (phase-phase, phase-amplitude, amplitude-phase, amplitude-amplitude). MI computation was performed using the direct method with quadratic extrapolation for bias correction in the Information-Theory Toolbox [33]. We quantised data into ten equi-populated bins but results were robust to changes in the number of bins. The result of this computation was a volumetric MI map (describing dependencies between speech and brain activity) for each frequency and individual. This computation was performed for the story condition and the back condition. In addition, surrogate MI maps were created by computing MI between the brain activity from the story condition and the reversed speech signal. This provides an estimate of MI values that can be expected by chance.

#### Statistics

Group statistical analysis was performed on the data of all 22 participants using non-parametric randomisation statistics in FieldTrip (Monte Carlo randomisation). Specifically, individual volumetric maps were smoothed with a 10 mm Gaussian kernel and subjected to dependent-samples T-test. The null distribution was estimated using 500 randomisations and multiple comparison correction was performed using FDR [74]. Only significant results (*p*<0.05 corrected) are reported. Group statistics were computed to compare the story condition to back condition and surrogate analysis. Final statistical maps (thresholded at *p*<0.05 corrected) are rendered on the MNI template brain. To confirm that MI for phase-phase interaction is due to phase-locking of speech and brain signals we computed PLV [75] and performed the same group statistics as for MI maps (Figure S1).

#### Lateralisation

Statistical analysis of lateralisation was performed in three steps. First, corresponding voxels in both hemispheres were identified on the basis of their coordinates. Second, the lateralisation index (LI = [right−left]/[right+left]) was computed for each voxel. Third, significance of lateralisation index was tested (*t*-test against 0) following the approach described in the previous paragraph with FDR correction for multiple comparisons. For Figure 3D we performed statistical comparison of theta lateralisation index against theta-gamma lateralisation index.

#### Complementarity of speech tracking mechanisms

To address the question whether MI I of theta speech phase (S_theta_) and theta brain phase (B_theta_) is significantly increased by including gamma amplitude in the computation (Figure 2D) we used the approach by Ince et al. [35]. The amount of information in gamma amplitude that is complementary to that of theta phase is computed as the difference of I (S_theta_, B_theta_ & B_gamma_) and I (S_theta_, B_theta_) using bias-corrected mutual information estimates (values are then expressed as percentage increase with respect to I [S_theta_, B_theta_]). The significance of the difference is tested by computing a null distribution without bias correction for I (S_theta_, B_theta_ & B_gamma_) where B_gamma_ is shuffled for fixed values of the binned signal B_theta_. The null distribution is then compared to I (S_theta_, B_theta_) computed without bias correction. These separate computations are motivated by the fact that bias correction decreases statistical power but increases accuracy of magnitude estimation [35].

#### Analysis of temporal speech edges

A thresholding algorithm was used to identify temporal edges in speech. The speech envelope was normalised to a maximum amplitude of 1. Speech edges were defined using the following criteria: (1) Mean amplitude in 400 ms before onset is less than 0.05. (2) Mean amplitude in 1 s after onset is larger than 0.05. (3) The difference between the mean amplitude 20 ms before and 20 ms after onset is larger than 0.05. For our particular speech stimulus this resulted in 254 time points characterised by a short period of low speech envelope amplitude followed by a sharp increase in amplitude. Onsets were confirmed by visual inspection of the speech envelope. Speech onset results were robust against small changes of these criteria. The same algorithm was applied to identify speech edges in the back condition. Mean and maximum amplitude and mean and maximum slope in the 100 ms following edge onsets were compared for the story and back condition and showed no significant difference (*t*-test, all *p*>0.05). Time-locked to these onsets we have extracted trials from −500 ms to 1,000 ms.

#### PLV analysis

PLVs [74] were computed in three ways. First, as phase-locking of auditory theta activity across trials (PLV = 1/*n*|∑ exp(i * ph)| where *n* is the number of trials and ph the phase of auditory theta signal). Second, the phase-locking of the phase difference between auditory theta signal and the theta speech envelope was computed (PLVsp = 1/*n* |∑ (exp(i * (ph−phs))| where *n* is the number of trials and ph the phase of auditory theta signal and phs the theta phase of speech envelope). Third, the phase-locking between left and right auditory theta activity (PLVsp = 1/*n* |∑ (exp(i * (phl−phr))| where *n* is the number of trials and phl and phr the phase of left and right auditory theta signal, respectively). Time-resolved PLV data were averaged in three time windows (−200 ms to 0 ms, 100–300 ms, 400–600 ms) and subjected to Anova analysis with factors time window and PLV measure. Both factors and their interactions were highly significant (time window: F = 39.77, *p*<0.001; PLV measure: F = 50.11, *p*<0.001; interaction: F = 14.86, *p*<0.001).

#### Speech sampling

For each voxel the instantaneous amplitude A and phase ph for each speech trial was computed (Figure 6). For each trial the cross-correlation of either cos(ph) or A with the speech envelope was computed over the time range 0–500 ms following onset with a maximum lag of 150 ms. The maximum correlation across lags was averaged across trials. As control the same computation was repeated with a random shuffling of trial order for the speech data (to destroy the correspondence between trials for speech and brain data).

#### Cross-frequency analysis

We performed two separate analyses to investigate the spatio-spectral distribution of cross-frequency coupling (Figure 7). First, we computed cross-frequency coupling between theta phase and 40 Hz gamma amplitude in all brain voxels. Second, we computed the full cross-frequency coupling matrix separately for the left and right auditory cortex.

The first analysis was motivated by Figure 2C that demonstrates coupling between speech theta phase and auditory 40 Hz amplitude dynamics and by Figure 4 that shows theta phase to gamma amplitude coupling in the auditory cortex. Analysis of cross-frequency coupling was performed by computing MI as in Figure 2C (but without using the speech signal). For each brain voxel MI between theta phase and gamma amplitude was computed for the two 500 ms windows preceding and following speech onset across all 254 trials. *t*-values of contrast post-onset versus pre-onset were computed across trials. The computation was performed for the story and back condition. As in Figure 2 individual maps were subjected to dependent samples *t*-test with randomisation-based FDR correction. Group *t*-maps are displayed with thresholds corresponding to *p*<0.05 (FDR corrected). The second analysis was performed only in the left and right auditory cortex. Here, we computed MI as before but now for all combinations of phase (range 1–10 Hz) and amplitude (range 4–80 Hz). Group *t*-statistic was computed for the difference between story condition and surrogate data (surrogate data were the same as story condition but each amplitude signal was matched with phase signal from a random trial).

For each frequency-frequency pair we computed a bootstrap confidence level by randomly drawing 22 participants with replacement in each of 500 bootstrap iterations and computing the 95th percentile.

The lateralisation analysis in Figure 7C follows the same approach as for Figure 7B and compares cross-frequency coupling for the story condition between the left and right auditory cortex.

## Supporting Information

Figure S1 (A) Mutual information group statistics for surrogate data. Group statistical map of phase-phase MI dependencies in the theta frequency band. This figure corresponds to Figure 2B but here the back condition has been replaced with a surrogate condition consisting of the MEG data from the story condition and the reversed speech envelope from the story condition to estimate dependencies that could be expected by chance. (B) Phase-locking group statistics. This figure corresponds to Figure 2 but instead of MI PLV has been used to quantify the dependence between phase of low-frequency speech envelope and brain activity in the delta band. (C) Same as (B) but for theta frequency band.
(PDF)

Figure S2 **Bar plot of individual lateralisation indices.** For each participant the lateralisation index for theta-phase lateralisation (red) and theta-gamma lateralisation (blue) in Heschl's gyrus (left panel) and superior temporal gyrus (STG, right panel) is shown. Each pair of red/blue bars corresponds to an individual.
(PDF)

Figure S3 **Bar plot of mutual information in the auditory cortex.** For each panel mean and SEM is shown for the left and right auditory cortex for all conditions. An asterisk indicates relevant significant differences (*t*-test with *p*<0.05). Control condition is computed from surrogate data where brain activity from story condition is used together with speech envelope from back condition. (A) Bar plot for delta phase. (B) Bar plot for theta phase. (C) Bar plot for mutual information between theta phase in speech and gamma amplitude in the auditory cortex. (D) Bar plot for mutual information between theta phase and gamma amplitude in the auditory cortex. Here, control condition was obtained from mutual information with gamma time series reversed.
(PDF)

Figure S4 **Group statistics of cross-frequency coupling.** (A) Statistical map of difference between story and back condition for mutual information between delta phase and theta amplitude. (B) Statistical map of lateralisation of mutual information between delta phase and theta amplitude for the story condition. (C) Statistical map of difference between story and back condition for mutual information between theta phase and gamma amplitude. This map corresponds to Figure 4A but is computed using a different method for quantifying cross-frequency coupling [76].
(PDF)

Figure S5 **Phase coding of speech amplitude.** The phase of theta oscillations at 100 ms after speech onset in the left (black) and right (red) auditory cortex codes the maximum amplitude of speech envelope in the first 200 ms following onset. The area signifies the 95% confidence interval around the median obtained from bootstrap analysis.
(PDF)

## Abbreviations

FDR: false discovery rate
MEG: magnetoencephalography
MI: mutual information
PLV: phase-locking value
