# Supplementary figures and images for: Speech Rhythms and Multiplexed Oscillatory Sensory Coding in the Human Brain
Source: PLoS Biol. 2013 Dec 31;11(12):e1001752. doi: 10.1371/journal.pbio.1001752 (PMC3876971; doi:10.1371/journal.pbio.1001752)

# A MI Surrogate

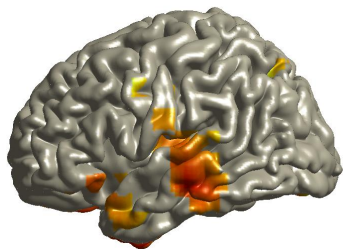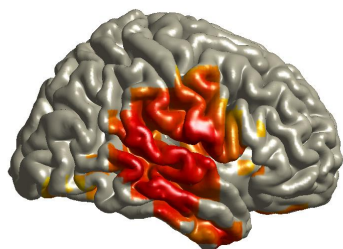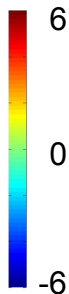

# B PLV delta

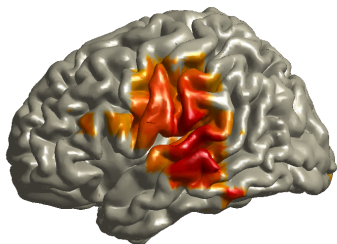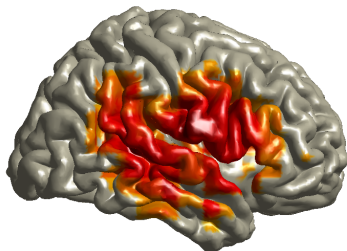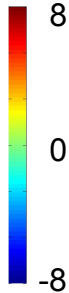

# C PLV theta

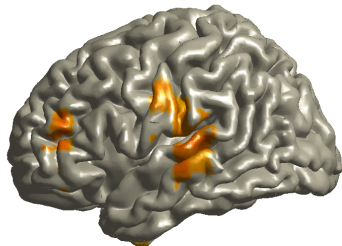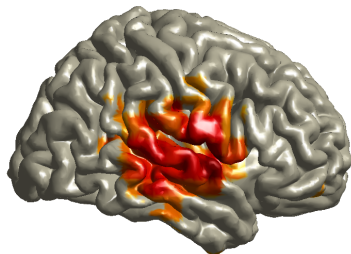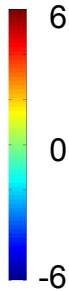

Supplement: Figure S1 — (A) Mutual information group statistics for surrogate data. Group statistical map of phase-phase MI dependencies in the theta frequency band. This figure corresponds to Figure 2B but here the back condition has been replaced with a surrogate condition consisting of the MEG data from the story condition and the reversed speech envelope from the story condition to estimate dependencies that could be expected by chance. (B) Phase-locking group statistics. This figure corresponds to Figure 2 but instead of MI PLV has been used to quantify the dependence between phase of low-frequency speech envelope and brain activity in the delta band. (C) Same as (B) but for theta frequency band. (PDF) [file pbio.1001752.s001.pdf]

Heschl

STG

right

0.8

-0.8

left

Subjects

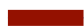

Theta Phase lateralisation

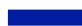

Theta-Gamma lateralisation

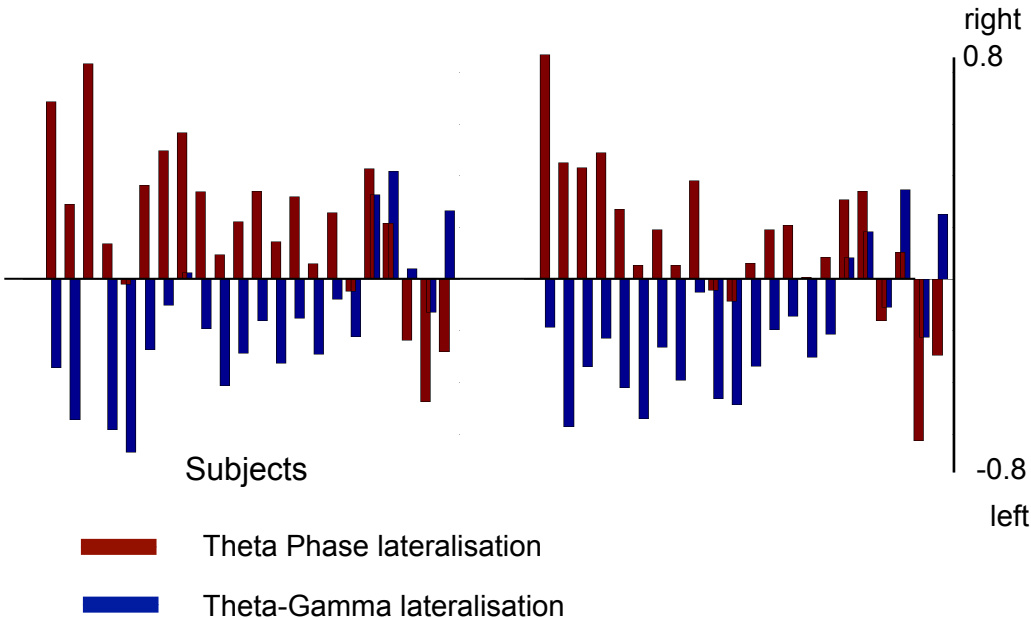

Supplement: Figure S2 — Bar plot of individual lateralisation indices. For each participant the lateralisation index for theta-phase lateralisation (red) and theta-gamma lateralisation (blue) in Heschl's gyrus (left panel) and superior temporal gyrus (STG, right panel) is shown. Each pair of red/blue bars corresponds to an individual. (PDF) [file pbio.1001752.s002.pdf]

**A delta**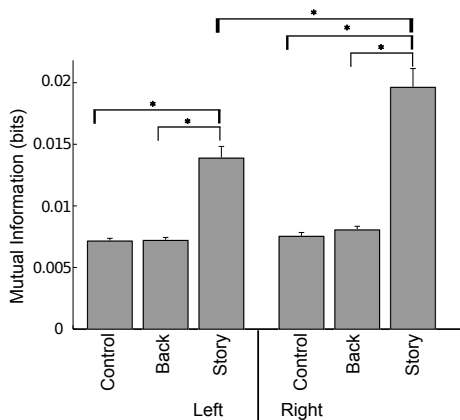**B theta**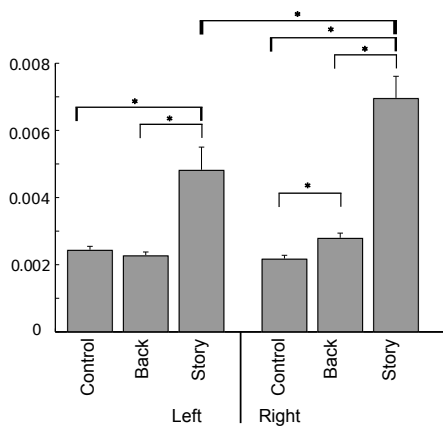**C theta gamma speech/brain**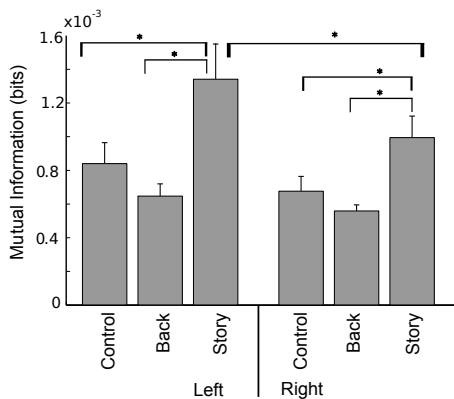**D theta-gamma brain**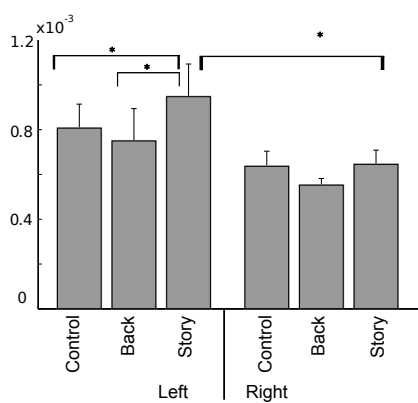

Supplement: Figure S3 — Bar plot of mutual information in the auditory cortex. For each panel mean and SEM is shown for the left and right auditory cortex for all conditions. An asterisk indicates relevant significant differences (t-test with p<0.05). Control condition is computed from surrogate data where brain activity from story condition is used together with speech envelope from back condition. (A) Bar plot for delta phase. (B) Bar plot for theta phase. (C) Bar plot for mutual information between theta phase in speech and gamma amplitude in the auditory cortex. (D) Bar plot for mutual information between theta phase and gamma amplitude in the auditory cortex. Here, control condition was obtained from mutual information with gamma time series reversed. (PDF) [file pbio.1001752.s003.pdf]

**A** Delta-Theta

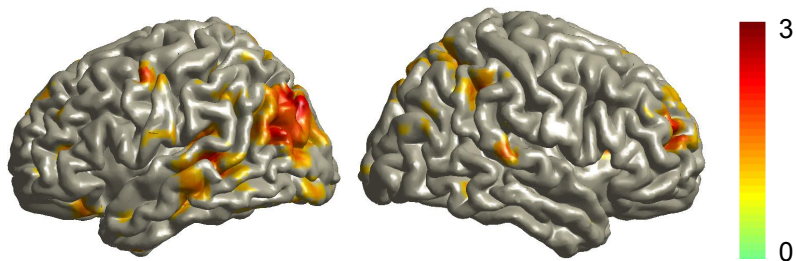

**B** Delta-Theta lateralization

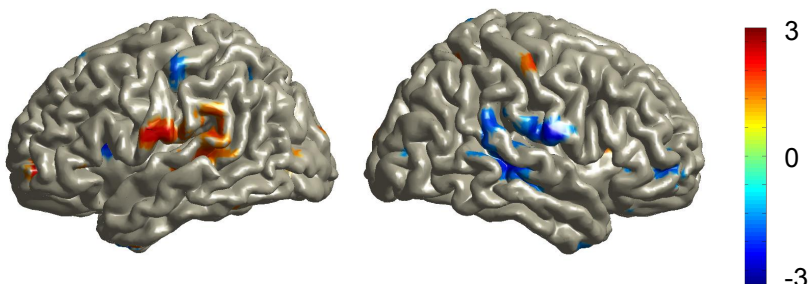

**C** Theta-gamma (alternative method)

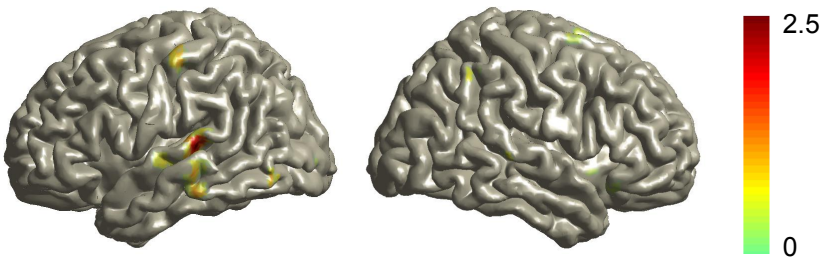

Supplement: Figure S4 — Group statistics of cross-frequency coupling. (A) Statistical map of difference between story and back condition for mutual information between delta phase and theta amplitude. (B) Statistical map of lateralisation of mutual information between delta phase and theta amplitude for the story condition. (C) Statistical map of difference between story and back condition for mutual information between theta phase and gamma amplitude. This map corresponds to Figure 4A but is computed using a different method for quantifying cross-frequency coupling [76]. (PDF) [file pbio.1001752.s004.pdf]

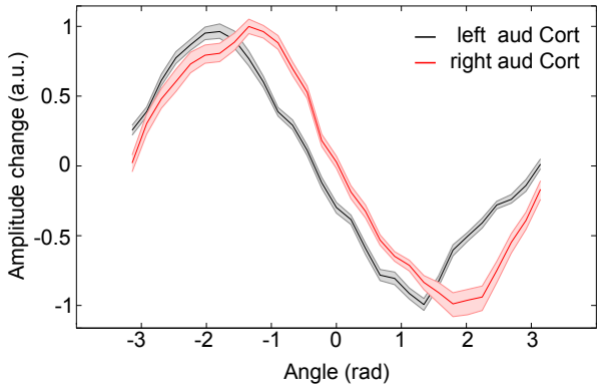

Supplement: Figure S5 — Phase coding of speech amplitude. The phase of theta oscillations at 100 ms after speech onset in the left (black) and right (red) auditory cortex codes the maximum amplitude of speech envelope in the first 200 ms following onset. The area signifies the 95% confidence interval around the median obtained from bootstrap analysis. (PDF) [file pbio.1001752.s005.pdf]
